# Knowledge of general practitioners about nasopharyngeal cancer at the Puskesmas in Yogyakarta, Indonesia

**DOI:** 10.1186/1472-6920-10-81

**Published:** 2010-11-18

**Authors:** Renske Fles, Maarten A Wildeman, Beni Sulistiono, Sofia Mubarika Haryana, I Bing Tan

**Affiliations:** 1 Department of Head and Neck Oncology and Surgery, The Netherlands Cancer Institute- Antoni van Leeuwenhoek Hospital, Plesmanlaan 121 1066 CX, Amsterdam, The Netherlands; 2 Department of Histology and Cell Biology Gadjah Mada University, Yogyakarta, Indonesia; 3 Molecular Biology laboratory, Gadjah Mada University, Yogyakarta, Indonesia; 4 Ear, Nose and Throat department, Gadjah Mada University, Yogyakarta, Indonesia

## Abstract

**Background:**

Nasopharyngeal carcinoma (NPC) is one of the leading causes of cancer death in Indonesia. At initial diagnosis, 80% of the patients present with advanced stage disease. In Indonesia, primary medical care is generally provided by the health care centres; named Puskesmas. The lack of knowledge of various aspects of NPC of the General practitioners (GPs) working in these centers might contribute to the diagnostic delay. The aim of this study was to assess the knowledge of these GPs on different aspects of NPC including symptoms, risk factors and incidence.

**Methods:**

One hundred six GPs in the Puskesmas in the Yogyakarta province were subjected to a questionnaire on different aspects of NPC based on literature and interviews with Head and Neck Surgeons.

**Results:**

All GPs approached participated and in total 106 questionnaires were filled in. All participants were aware of NPC as a disease and 89% confirmed that it is a serious problem in Indonesia. However, 50% of the participants believed NPC has a low incidence in their region. The question on early symptoms gave a mean 4.2 answers of which 50% were incorrect.

The GPs provided a total of 318 answers when asked for the risk factors of NPC, 75% of which were incorrect. Fifty seven GPs (54%) stated that they did not receive sufficient education on NPC at the university and insufficient knowledge was gained during daily practice. Ninety-two percent of the GPs were interested in additional education, preferably in form of lectures, meetings or folders.

**Conclusion:**

This study revealed that GPs in the Puskesmas in Yogyakarta lack knowledge on all aspects of NPC. This is an important finding as NPC is endemic in Indonesia and the Puskesmas are the institutions which provide primary medical health care in the country. Further education of the GPs in these endemic areas could be a first step to increase the rate of early detection. Therefore, we suggest 1) to conduct a medical awareness campaign for GPs on the most important subjects concerning NPC, and 2) as soon as NPC awareness among GPs has risen, provide further education on the risk factors, the early symptoms and the incidence, education to the community. We propose to extend this study to other areas in Indonesia (i.e. Jakarta, Surabaya, Central Java), using models that have been developed in Yogyakarta.

## Background

NPC is a rare malignancy in most parts of the world. In year 2000 there were approximately 65,000 new cases and 38,000 deaths worldwide [1]. However, NPC is endemic in a few of well-defined populations, for example in China, Southeast Asia, Arctic, Middle East/North Africa and North America [2]. The highest incidence has been observed in Hong Kong where approximately 1 out of 40 men developed a NPC before the age of 75 [3].

NPC in Indonesia has a relatively high incidence of at least 5.7 among men and 1.9 among women per 100.000 compared to the worldwide incidence 1.9 among men and 0.8 among women (http://www.iarc.fr). It should be noted that the actual incidence of NPC in Indonesia is unknown due to poor cancer registries. For examples, the Ear, Nose and Throat (ENT) department of the Dr. Sardjito Hospital in Yogyakarta already has 200 new cases of NPC a year and is 1 of the 4 big hospitals in Yogyakarta (data of unpublished study).

One of the most important factors playing a role in the tumour development of NPC is the presence of the Epstein-Barr virus (EBV). The undifferentiated carcinoma (WHO type III) is always associated with EBV [4]. In addition, high levels of volatile nitrosamines in preserved food can be found in the high incidence regions, and has shown to be a putative carcinogen for the development of NPC [5]. Also, the consumption of salty-preserved fish has been strongly associated with an increased risk of developing NPC [6-12]. The non-environmental risk factors include: gender, ethnicity and the family history.

The non-specific nature of the early symptoms makes NPC a disease challenging to diagnose at early stage. The most common presenting symptom is a painless mass in the neck. Early symptoms like nasal dysfunction, aural dysfunction and headache are non-specific for NPC but should be a warning sign in endemic areas. Given these non-specific of NPC, 80% of the patients present themselves with a stage 3 or 4 disease.

The choice of treatment for primary NPC is radiotherapy, with chemotherapy for advanced stage disease. Despite the radio responsiveness of nasopharyngeal tumours, good long-term survival is only achieved for patients with early disease and with minimal neck involvement [13-15].

Given that prognosis is better when treatment is started at an earlier stage of the disease, it is of major importance to determine the various factors that may possibly contribute to the diagnostic delay of NPC in Indonesia.

We hypothesized that one of the possible mechanisms leading to late NPC presentation observed in Indonesia is a lack of knowledge and awareness among primary health care workers; the GPs working in the primary health care centres; Puskesmas.

In this study we aimed to assess the current knowledge amongst GPs in the Puskesmas in Yogyakarta about NPC regarding risk factors, incidence and symptoms. The findings from this study will be useful to complement knowledge and awareness on this important public health issue in Indonesia.

## Methods

### Interview and questionnaire development

A questionnaire based on literature was designed appointing the most important aspects of NPC; including the risk factors, early symptoms, as well as the education possibilities and the interest in education. This first draft was discussed during a semi constructed interview with four Head and Neck surgeons from Universitas Indonesia, Jakarta, Universitas Gadjah Mada, Yogyakarta and The Netherlands Cancer Institute, Amsterdam. The questionnaire was adapted according to consensus opinions.

The second draft was filled in by 10 Indonesian ENT-specialist who gave oral and written comment. These ENT specialists listed the key aspects about NPC a GP should be familiar with, which include the awareness of early NPC symptoms and how to pursue if a patient shows these symptoms. GPs are often unaware to whom and when they should refer a patient. If they refer, patients are often referred to a general surgeon rather than to an ENT specialist.

### Questionnaire

The questionnaire consists of four sections, assessing (1) general information about the GPs such as number of years of experience and the number of patients seen in one year; (2) knowledge on NPC such as early symptoms and risk factors (3) incidence, experience in daily practice regarding the extent to which the GPs had been confronted with NPC in their Puskesmas and how they dealt with patients suspected of NPC; and (4) ambition and wishes regarding future education on NPC.

The questionnaire contained open and closed questions. Possible responses were grouped together for the ease of presentation and understanding as described in the results. The open-ended questions featured the questions where multiple answers were possible, for instance when asked about risk factors or presenting symptoms.

### Study population

The study population consisted of GPs from three different districts in the Yogyakarta province. In total there are 68 Puskesmas in these regions. With two or three GPs working in one Puskesmas, approximately 170 GPs are practicing in a Puskesmas in this area. One hundred and six GPs were approached in their Puskesmas to participate. They were asked to fill in the questionnaire directly in the presence of the researcher (RF).

## Results

All 106 GPs approached participated in this study and completed their questionnaires (100%). All participants know NPC as a disease and 89% confirmed it is a serious problem. Fifty-nine questionnaires were completed immediately during the visit of the interviewer. The other forty-seven were completed at a later time point. Patients waiting for medical attention, was the reason for completion questionnaire without the presence of the researcher. Thirty questionnaires were collected in the centre of Yogyakarta (Kota), the other 76 questionnaires were collected in the rural areas, Sleman and Bantul. The number years of work experience of the GPs varied from less than one year to 29 years (median = 5 years). There was no correlation between working experience and knowledge on NPC. Participant details are outlined in Table 1.

**Table 1:** Descriptive divided by the three district of the province Yogyakarta

Questions		Kota (n = 30)	Sleman n = 42)	Bantul (n = 34)
**How long have you worked as a doctor? (in years)**		8.2	8.1	6.7

**Have you always worked in this area?**	**yes**	26	42	32
	
	**no**	3	0	2
	
	**missing**	1	0	0

**From which university did you get you medical degree?**	**UGM**	18	26	21
	
	**other**	12	16	12

**How many patients do you treat a year?**		10364	7944	6236

**How many GP's are practicing in your Puskesmas?**		2.9	2.8	2.9

Overview of the descriptive variables over the three districts.

### Knowledge about the early symptoms and risk factors

When asked to list early symptoms on NPC, the participating GPs gave a mean of 4.2 answers. However, 51% of these answers were incorrect (Table 2). The most frequent given answer provided was swelling in the neck (n = 78; 35%). Nasal obstructions and epitaxis, caused by presence of tumour mass in the nasopharynx, were mentioned 70 times (32%). Tinnitus and hearing loss due to dysfunction of the Eustachian tube, associated with the lateroposterior extension of the tumour to the paranasopharyngeal space was stated 33 times (15%). Headache, diplopia, facial pain and numbness was pointed out 25 (11%) times, these symptoms are caused by skull-base erosion and palsy of the fifth and sixth cranial nerves, associated with the superior extension of the tumour.

**Table 2:** Number of correct and incorrect answers.

	correct	incorrect	total
**symptoms**	227 (51%)	215 (49%)	442

**risk factors**	113 (36%)	203 (64%)	316

Overview of the total numbers of correct and incorrect answers when asked about the symptoms and the risk factors of NPC.

Of the 106 participants only 20 participants provided four or more correct symptoms of NPC. The majority of the GPs (66%) could give two correct answers. The most frequent incorrect NPC symptoms stated answers were hoarseness and dysphagia.

The risk factor questions yielded a total of 328 answers, of which only 36% were indeed risk factors for NPC (Table 2). Surprisingly, EBV was only mentioned 12 times, while smoking, which is not a risk factor for NPC in Indonesia, was answered 80 times. Seven out of the eight participants who provided only one answer gave an incorrect answer.

### Knowledge on incidence and referral

Fifty percent of the participating GPs falsely believed that NPC has a low incidence in their region. Furthermore, only 45% was aware of the fact NPC can affect both children and adolescents (table 3). Seventy four percent of the GPs stated that they had never seen a patient with NPC in their Puskesmas. Enlarged lymph nodes is the most common presenting symptom for NPC. The differential diagnosis for these lymph node enlargements is limited and in most cases related to tumours in the ENT region. Strikingly, 80% of the GPs have seen patients with enlarged lymph nodes in the daily practice. In an open question on referral of a suspected NPC case, only 18% percent would refer the patient to an ENT specialist. Further details are shown in table 4.

**Table 3:** General knowledge of the General Practitioners about NPC in Indonesia

Have you ever heard of nasopharyngeal carcinoma?	yes	no				missing
	106 (100%)	0				0

**NPC is a serious problem in Indonesia/Java**	**strongly** **agree**	**agree**	**neutral**	**disagree**	**Strongly** **disagree**	**missing**

	19 (18%)	73(69%)	9 (8%)	2 (2%)	0	3 (3%)

**The number of new people suffering NPC on Indonesia per year**.	**10-99**	**100-249**	**250-499**	**500-999**	**> 1000**	**missing**

	6 (6%)	17 (16%)	21 (20%)	9 (8%)	25 (24%)	28 (26%)

**From what age people can develop NPC?**	**0-19**	**20-29**	**30-39**	**40-49**	**> 50**	**missing**

	8 (8%)	39 (37%)	37 (35%)	15 (14%)	0	7 (7%)

**Which age category has the highest incidence?**	**0-19**	**20-29**	**30-39**	**40-49**	**> 50**	**missing**

	1 (1%)	3 (3%)	35 (33%)	49 (46%)	10 (9%)	8 (8%)

**Do you know what the difference in incidence is between men and women?**	**1:1**	**1:2**	**2:1**	**1:3**	**3:1**	**missing**

	3 (3%)	7 (7%)	51 (48%)	4 (4%)	30 (28%)	11 (10%)

General questions about NPC concerning the incidence, severity, age and relation to gender.

**Table 4:** Question related to the situation in the puskesmas

Did you ever treat a patient with NPC?	yes	no				missing
	16 (15%)	89 (84%)				1 (1%)

**Have you ever seen a patient with NPC in your clinic?**	**never**	**< 2times per year**	**2-5 times per year**	**5-10 times per year**	**> 10 times**	**missing**

	78 (74%)	22 (20%)	4 (4%)	0 (0%)	1 (1%)	1 (1%)

**The number of new people suffering NPC in your region per year?**	**< 100**	**100-250**	**250-500**	**500-1000**	**> 1000**	**missing**

	80 (75%)	0 (0%)	2 (2%)	0 (0%)	1 (1%)	23 (22%)

**Have you seen a shift in the number of people suffering NPC in year clinic the last year?**	**yes, more patients**	**yes, less patients**	**no**			**missing**

	7 (7%)	2 (2%)	91 (86%)			6 (6%)

**How often do you, in average a year, treat patients with enlarged neck lymph nodes?**	**never**	**< 2times per year**	**2-5 times per year**	**5-10 times per year**	**> 10 times**	**missing**

	21 (20%)	32 (30%)	30 (28%)	13 (12%)	6 (6%)	4 (4%)

**How did you treat most of the patients?**	**No treatment**	**medication**	**local surgery**	**refer to hospital**	**medication and referring to a hospital**	**missing**

	4 (4%)	15 (14%)	1 (1%)	62 (58%)	13 (12%)	10 (9%)

**What do you do when you diagnose a patient with NPC?**	**refer to hospital**	**refer to academic hospital**	**refer to ENT specialist**	**refer to specialist**	**refer if no improvement of treatment**	**missing**

	58 (55%)	3 (3%)	13 (12%)	1 (1%)	1 (1%)	30 (28%)

Questions related to the frequency of patients with NPC recognized by the GP, treatment and the referring system of the GP

When GPs were asked if they would be interested in additional education on NPC; 92% of the participants responded positive, 7% were neutral and only one participant strongly disagreed. Although the differences between the preferences for the way to educate were small, 96% stated a lecture from an ENT-specialist is the best form of further education on NPC. A good alternative or addition could be a folder (88%). Eighty two percent believes that receiving credits and a certification for participation is important. Finally, 40% of the participants recommended a refreshing course on NPC in the open comment box.

## Discussion

Our study revealed that knowledge on essential aspects of NPC among GPs in the Puskesmas in Yogyakarta, Indonesia is poor. This finding is of importance as NPC is endemic in Indonesia and the Puskesmas are institutions, which provided primary medical care in the country. This lack of basic knowledge of NPC might be a fundamental cause for late stage discovery of the disease by GPs.

The majority of the doctors believed that the incidence of NPC in their region is at least a tenfold lower than the estimated incidence. Together with the lack of knowledge on early NPC symptoms and risk factors we can conclude that the knowledge about NPC of these doctors is not sufficient.

Enlarged lymph nodes in the neck is the most common symptom of NPC at presentation. The participants of this study regularly see patients with enlarged neck lymph nodes, but stated they see very few cases of NPC. Combining the overall answers given regarding early symptoms and incidence, we think GPs often do not consider NPC when they see a patient with enlarged neck lymph nodes or other symptoms suspicious for NPC.

A possible solution could be education combined with a screening method. Fachiroh et al has established a cheap and sensitive screening method for NPC which could be easily adopted in the Puskasmas [16]. This method is based on the detection of the Epstein-Barr virus which is present in all NPCs in Indonesia. The combination of more knowledge and an easy way to screen might lead to early detection of NPC.

Following the answers provided by the participants, the number of NPC patients seen by the GPs is much lower compared to the incidence in this region. The results of our study imply that this may be due to the fact that GPs are unable to identify patients with NPC. It should be noted that the majority of the patients, who were treated at the ENT-departments either went directly to a hospital without referral by a GP, or were administered to a hospital as a medical emergency caused by the tumour, for example dyspnoea to massive neck metastasis. In an ongoing study we look into more detail at the referral system.

### Study Limitations

Not all questionnaires were completed in the presence of the researcher. This was mostly due to lack of time on the GPs side as patients were waiting for their appointments. Despite the statement in the questionnaire not to seek help from third parties, GPs may have searched or asked colleagues for answers in our absence. The results of these 47 participants, who filled in the questionnaires in the absence of the interviewer, were notable better but overall remained poor. For example, 9 of the 12 GPs who knew that EBV was related to NPC completed the questionnaire without supervision. This could imply that even if GPs would have searched for information elsewhere the correct information was not available.

In addition, some of the questions despite our testing may not have been understood completely by the participating GPs. For instance, there was a big variance between the answers provided for the number of patients seen a year, even if these doctors worked in the same Puskesmas. For example, in one Puskesmas two GPs stated that they see 18.000 patients a year, whilst the other GP only sees 4000 patients a year.

## Conclusion

Based on the results of this survey it is clear that the knowledge of the GPs about NPC in the Puskesmas in Indonesia is not sufficient to deal with this important health issue. The problem concerning the identification of patients suffering from NPC is most likely bigger than expected. The number of patients with NPC registered in Sardjito Hospital in the period of 2004 and 2008 might only represent the tip of the iceberg since these are the patients who actually made it to the hospital.

A medical education program should be started to educate the GPs working in the Puskesmas in Indonesia about NPC. The next step could be a campaign to increase public awareness on NPC. Similar campaigns have been enrolled for cervix carcinoma and breast carcinomas [17]. The public needs to be informed to visit the Puskesmas when they have complaints or early NPC symptoms.

## Competing interests

The authors declare that they have no competing interests.

## Authors' contributions

RF obtained the questionnaires and interviews, participated in study design and the analyses and in writing the manuscript. MAW conceived the study, participated in the study design and development of the questionnaire, participated in the analyses and in writing the manuscript. RF and MAW contributed equally to the production of this manuscript. BS participated in the coordination of the study. IBT and SMH participated in the analyses and writing of the manuscript. All authors read and approved the final manuscript.

## Pre-publication history

The pre-publication history for this paper can be accessed here:

http://www.biomedcentral.com/1472-6920/10/81/prepub
